# Supplementary material for: Automated curriculum learning for embodied agents a neuroevolutionary approach
Source: Sci Rep. 2021 Apr 26;11:8985. doi: 10.1038/s41598-021-88464-5 (PMC8076209; doi:10.1038/s41598-021-88464-5)
Supplement: Supplementary file 1 — Supplementary Information [file 41598_2021_88464_MOESM1_ESM.pdf]

Automated Curriculum Learning for Embodied Agents
A Neuroevolutionary Approach

Nicola Milano and Stefano Nolfi
Institute of Cognitive Science and Technologies,
National Research Council, Roma, Italy
nicola.milano@istc.cnr.it, stefano.nolfi@cnr.it

Table 1 and 2 report the p-value for the pairwise comparison among all the conditions in the case of the long double-pole and bipedal walker hardcore problems

Table 1. P-values from the Mann-Whitney U test with Bonferroni correction α = 5 for the long double-pole problem.

	standard	linear	X^2	X^3	X^4
Standard	-	0.008	0.004	$2*10^4$	$7*10^4$
Linear	-	-	0.007	$8*10^4$	0.004
X^2	-	-	-	0.005	0.08
X^3	-	-	-	-	0.006
X^4	-	-	-	-	-

Table 2. P-values from the Mann-Whitney U test with Bonferroni correction α = 5 for the bipedal walker hardcore problem.

	standard	linear	X^2	X^3	X^4
Standard	-	0.007	0.005	0.004	0.003
Linear	-	-	0.007	0.006	0.006
X^2	-	-	-	0.06	0.08
X^3	-	-	-	-	0.1
X^4	-	-	-	-	-
